# The synchronous TAG production with the growth by the expression of chloroplast transit peptide-fused ScPDAT in *Chlamydomonas reinhardtii*

**DOI:** 10.1186/s13068-018-1160-6

**Published:** 2018-06-06

**Authors:** Zhen Zhu, Guangze Yuan, Xuran Fan, Yan Fan, Miao Yang, Yalei Yin, Jiao Liu, Yang Liu, Xupeng Cao, Jing Tian, Song Xue

**Affiliations:** 1 School of Bioengineering, Dalian Polytechnic University, Dalian, 116034 China; 2 Marine Bioengineering Group, Dalian Institute of Chemical Physics, Chinese Academy of Sciences, Dalian, 116023 China; 3 University of Chinese Academy of Sciences, Beijing, 100049 China; 4 Key Laboratory of Separation Science for Analytical Chemistry, Scientific Research Center for Translational Medicine, Dalian Institute of Chemical Physics, Chinese Academy of Sciences, Dalian, 116023 China

**Keywords:** Phospholipid: diacylglycerol acyltransferase, PDAT, Synchronous TAG production, *Chlamydomonas reinhardtii*, Lipid

## Abstract

**Background:**

The synchronous triacylglycerol (TAG) production with the growth is a key step to lower the cost of the microalgae-based biofuel production. Phospholipid: diacylglycerol acyltransferase (PDAT) has been identified recently and catalyzes the phospholipid contributing acyl group to diacylglycerol to synthesize TAG, and is considered as the important source of TAG in *Chlamydomonas reinhardtii*.

**Results:**

Using a chimeric Hsp70A–RbcS2 promoter, exogenous PDAT form *Saccharomyces cerevisiae* fused with a chloroplast transit peptide was expressed in *C. reinhardtii* CC-137. Proved by western blot, the expression of ScPDAT showed a synchronous trend to the growth in the exponential phase. Compared to the wild type, the strain of *Scpdat* achieved 22% increase in the content of total fatty acids and 32% increase in TAG content. In addition, the fluctuation of C16 series fatty acid in monogalactosyldiacylglycerol, diacylglyceryltrimethylhomoserine and TAG indicated an enhancement in the TAG accumulation pathway.

**Conclusion:**

The TAG production was enhanced in the regular cultivation without the nutrient stress by strengthening the conversion of polar lipid to TAG in *C. reinhardtii* and the findings provide a candidate strategy for rational engineered strain to overcome the decline in the growth during the TAG accumulation triggered by nitrogen starvation.

**Electronic supplementary material:**

The online version of this article (10.1186/s13068-018-1160-6) contains supplementary material, which is available to authorized users.

## Background

As a promising source of biodiesel, microalgae have also been a good model to investigate the biological process of triacylglycerol (TAG) production [1]. Aiming to harvest more TAG, stress conditions were normally applied in the cultivation of microalgae. However, the overall TAG productivity was limited due to the inhibition of the growth under the stress conditions, especially nutrient depletion conditions. Even numerous trials have been carried out; it is still far from success to achieve the synchronized TAG production with cell growth, which is generally considered as an essential feature of the ideal strategy for economical TAG production.

To improve the TAG production, besides the engineering approach, another way is to modify fatty acids and lipids’ metabolism flux relating to TAG synthesis by molecular biological methods, e.g., mutating key enzymes, reducing or increasing the expression and introducing exogenous genes. Till now, widely concerned targets include the genes of acyltransferases such as glycerol-3-phosphate acyltransferase (GPAT, EC 2.3.1.15), lysophosphatidic acid acyltransferase (LPAAT, EC 2.3.1.51), acyltransferase: diacylglycerol acyltransferase (DGAT, EC 2.3.1.20) [2–6] and the phospholipid: diacylglycerol acyltransferase (PDAT, EC 2.3.1.158) [1]. Among the above acyltransferases, PDAT was the only non-CoA-dependent acyltransferase synthesized using phospholipid acyl as donor, which was first identified in the castor by Anders and his colleagues in 2000 [7]. Before that, DGAT was considered the only enzyme that synthesizes TAG from DAG [8] and the acyl-CoA was an essential in the synthesis process.

Based on the discovery of PDAT, more researchers are focusing on the conversion of polar lipids to TAG, and the role of PDAT during the TAG production (Table 1). For instance, Zhang et al. [9] reported that PDAT1 is essential for normal pollen and seed development in *Arabidopsis*. In microalgae, the first PDAT was identified in *C. reinhardtii* (CrPDAT) and was reported with broader substrate specificity and distinct lipase functions by Yoon et al. [10]. CrPDAT not only hydrolyzes glycolipids, phospholipids and TAG, but also hydrolyzes cholesterol esters [similar to lecithin–cholesterol acyltransferase’s (LCAT) function] with a preference to use anionic phospholipids (PA/PS/PI/PG) as the acyl donor in vitro [10]. On the contrast, PDATs of other species, including LuPDAT/ScPDAT/AtPDAT, are more likely to utilize cationic phospholipids (PC/PE) as acyl donors [1, 11, 12]. However, the preference of PDAT to diacylglyceryltrimethylhomoserine (DGTS) as the donor, which is the replacement of PC in *C. reinhardtii*, has not been known by far.

**Table 1:** A functional study of PDAT derived from different species

Gene	Origin	Research	Conclusion	References
CrPDAT	*Chlamydomonas reinhardtii*	Utilizing RNA-Seq insert mutants Artificial microRNA silencing of PDAT	Demonstrating the relevance of the transacylation pathway CrPDAT possesses acyl hydrolase activities, mediated membrane lipid turnover and degradation	[13] [10]
ScPDAT	*Saccharomyces cerevisiae*	Lacking the predicted membrane-spanning region of ScPDAT expressed in *Pichia pastoris*	ScPDAT can catalyze a number of transacylation reactions at a low rate	[11]
AtPDAT	*Arabidopsis*	Exploring role of enzymes in TAG synthesis by RNA interference Over-expression of AtPDAT in microsomal preparations of roots and leaves Coexpression of PDAT1 with oleosin Disruption of SDP1 TAG lipase or PXA1 severely decreases FA turnover, leading to increases in leaf TAG accumulation	The velocity of AtPDAT dependent on acyl composition Enhancing fatty acid synthesis and diverting fatty acids from membrane lipids to triacylglycerol Over-expression of PDAT1 enhances the turnover of FAs in leaf lipids	[9] [12] [14] [15]
LuPDAT	*Linum usitatissimum* L.	Over-expression of LuPDAT genes in yeast and *Arabidopsis*	Certain PDATs have the unique ability to efficiently channel ALA into TAG	[16]
MiPDAT	*Myrmecia incisa* Reisigl	Over-expression of *MiPDAT* and increase transcription levels in *M*. *incisa*	The mechanism is discussed that MiPDAT in this microalgal uses PC to yield TAG	[1]

**Fig. 1:**
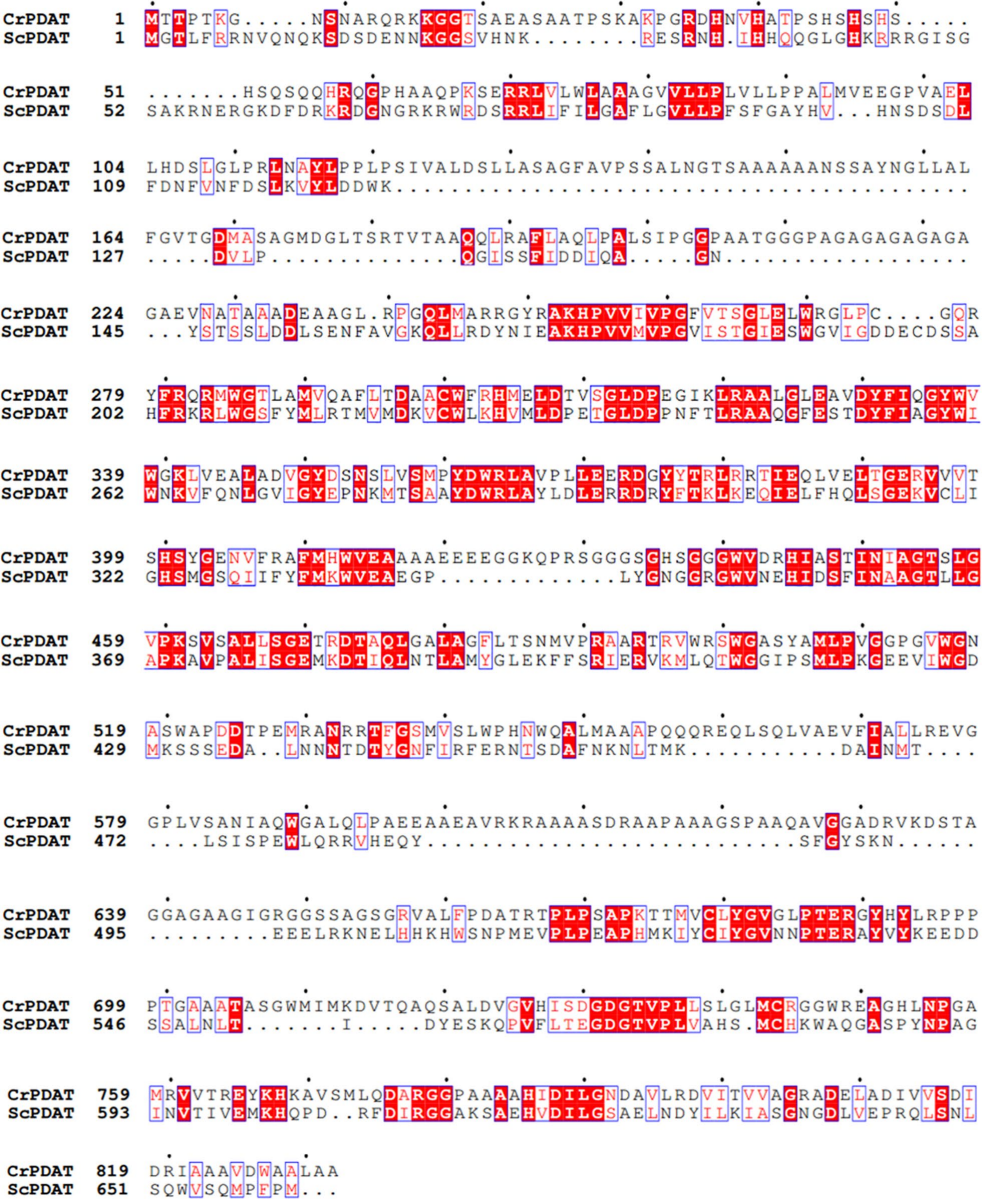
The sequence alignment of de-gapped CrPDAT and ScPDAT. The protein sequences of CrPDAT (GenBank: AFB73928) without gap1 and gap2 regions and ScPDAT (GenBank: NM_001183185) were aligned by Clustalw2 and Espript 3.0 software

While comparing the sequence of PADT in *C reinhardtii* CC-137 to the other PADTs’, a high similarity between PDAT from *Saccharomyces cerevisiae* (ScPDAT) and CrPDAT was shown with 35% similarity [10]. The major difference of CrPDAT to ScPDAT lies in the existence of two unique gap regions, GAP1 (570–700) with 14 consecutive SG repeats (black underlined in Fig. 1), GAP2 (840–930) with 30 amino acid residues A and 13 G (dotted line in Fig. 1). However, the function of these gaps is unknown. Setting aside gaps, ScPDAT shares more than 40% similarity to CrPDAT (Additional file 1: Figure S1). Based on the alignment analysis, ScPDAT can be considered as a CrPDAT without gaps and may act instead of CrPDAT while over-expressed in *C. reinhardtii* to inspect its function as acyltransferase.

To explore the role of PDAT during the TAG accumulation, ScPDAT was used and over-expressed in *C. reinhardtii*. Especially, taking into consideration that CrPDAT is located in the chloroplast of *C. reinhardtii*; the chloroplast transit peptide (cTP) of CrPDAT was fused in the front of ScPDAT. Here we started from the over-expression of cTP-fused ScPDAT in *C. reinhardtii* CC-137 and inspected the difference between engineered strain and wild type, on the effects on the accumulation of the lipids and fatty acid profile. The results will be helpful in understanding PDAT’s bio-functions and contribute to the theoretical foundation of TAG production in microalgae.

## Results and discussion

### Verification of ScPDAT sequence and transformants

**Fig. 2:**
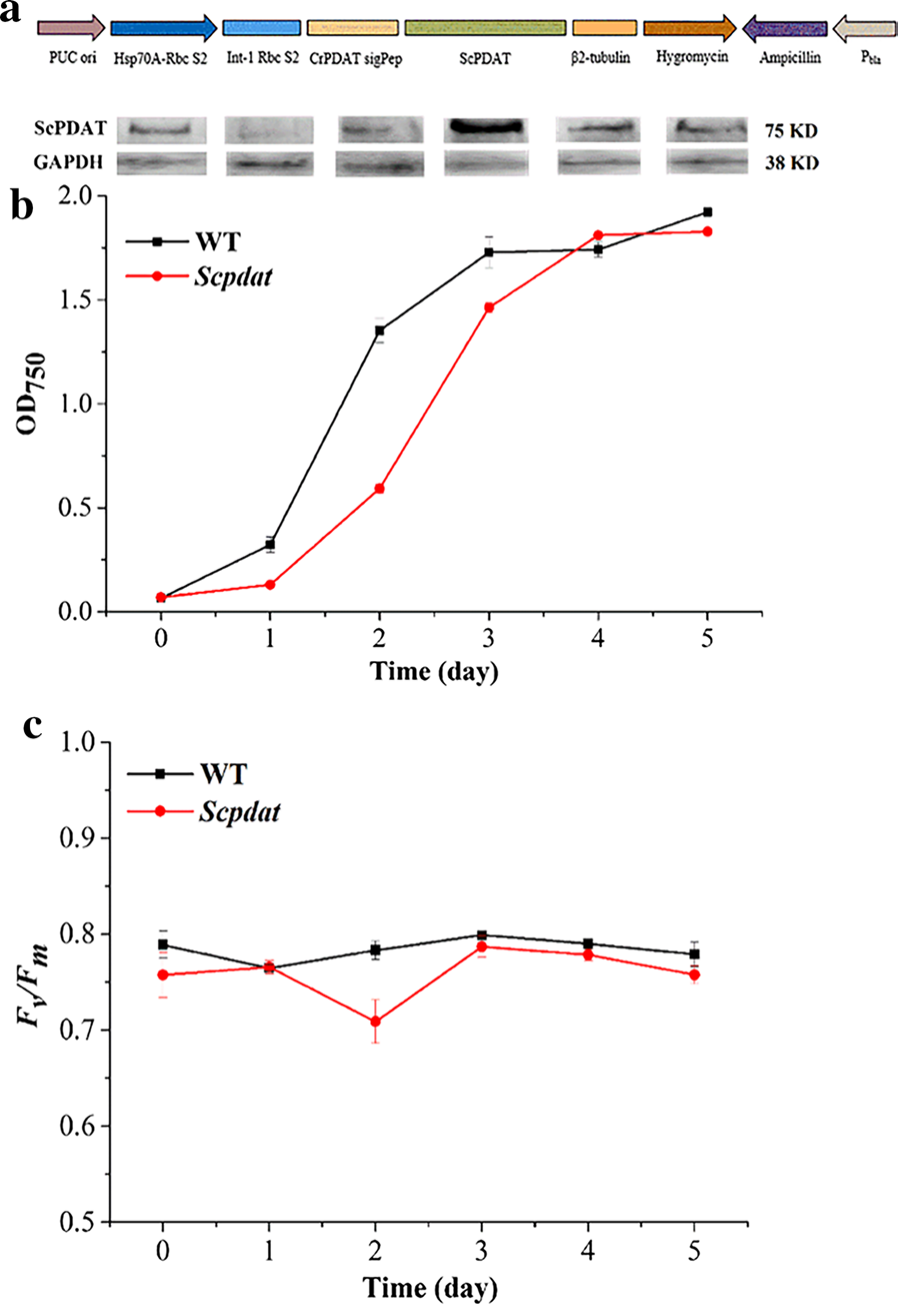
The construction and effects of exogenous ScPDAT. **a** The map of pChlamy–ScPDAT plasmid. PUCori: (high-copy replication and growth in *E. coli*, 673 bp), Hsp70A–RbcS2 (a hybrid constitutive promoter, 495 bp), Int-1 Rbc S2 (maintains the high expression of ScPDAT, 144 bp), β2-tubulin (drives the expression of Aph7 gene, 312 bp), Hygromycin (selection of *C. reinhardtii*, 1310 bp*),* Ampicillin (selection of the plasmid in *E. coli*, 860 bp), Pbla (expression of the ampicillin resistance gene, 51 bp). **b** Western blot of ScPDAT in *Scpdat*, the growth curve of wild type and *Scpdat*. Cells sampled during a 5-day culture cycle and grown in TAP medium. Each type of algae takes three bottles a day and does not put them back. Shown are mean values and SD; *n *= 3. Assay conditions are described in “Methods”. **c** The chlorophyll fluorescence of wild type and *Scpdat*. Shown are mean values and SD; *n *= 3. Assay conditions are described in “Methods”

The ScPDAT and a pChlamy vectors were integrated by RF cloning to construct the pChlamy–ScPDAT recombinant plasmid. The results are shown in supplementary materials (Additional file 2: Figure S2A). After cloned into *Escherichia coli* DH5α, the plasmid was sequenced and verified as the design (Fig. 2a).

After electroporated into *C. reinhardtii* CC-137, the transformants of pChlamy–ScPDAT were selected and verified by direct PCR amplification (Additional file 2: Figure S2B). Randomly, nine positive clones were inoculated into 100-mL shake flasks and no obvious difference in the growth was observed and one strain was selected and named as *Scpadt* for the further investigation.

### The growth and chlorophyll fluorescence variation of *Scpadt*

The growth and *F*_v_/*F*_m_ in *Scpdat* was monitored and compared with those of the wide type (WT). *Scpdat* showed a slow initial growth on the first day (Fig. 2b), together with a relatively lower *F*_v_/*F*_m_ (Fig. 2c). Then the growth turned into the exponential phase till the fourth day and achieved the same final OD_750_ as WT. There was also no significant difference in the final biomass, about 0.80 g/mL (dry weight).

For *F*_v_/*F*_m_, both WT and *Scpdat* showed a slight decrease at the beginning of the exponential phase, in the first and second day, respectively, and *Scpdat* decreased more than WT. Then both strains recovered and kept the similar trend while *Scpdat* remained 0.1–0.2 unit lower than WT.

### Expression of ScPDAT in *Scpadt*

The expression during the above 5 day’s cultivation was monitored by western blot and GAPDH was used as the control. The expression of ScPDAT changed significantly during the cultivation. The protein expression reached to the peak on the third day, and then fell down to a stable level (Fig. 2b). It is notable that the expression of ScPDAT increased consistently with the growth within the exponential phase (Fig. 2b).

### Fatty acid profile and total lipid content

**Fig. 3:**
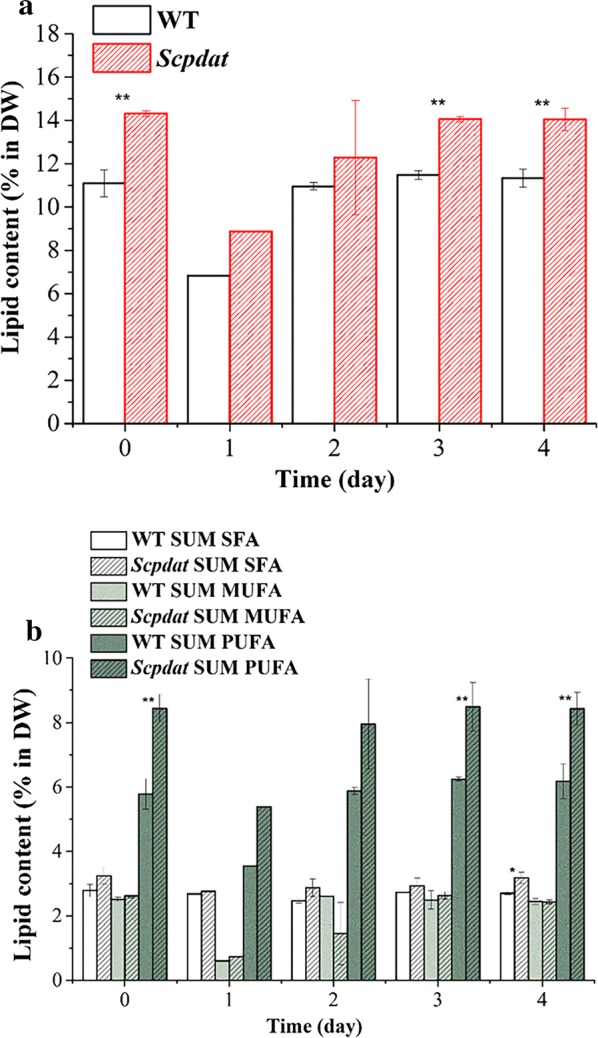
The effects on FAs of *Scpdat*. **a** The total lipid content in dry weight of the wild type and *Scpdat*. During the culture period of 0–4 days, the left and right columns in each day represent the wild type and *Scpdat*, respectively. Dates are shown as * and ** denote significant difference (*p* < 0.05) and extremely significant difference (*p* < 0.01), respectively. **b** The contents of SFA, MUFA and PUFA in wild type and *Scpdat*. The fatty acids of wild type and Scpdat were classified into three categories: SFA, MUFA, PUFA, and compared with the content for each kind of fatty acid, respectively. Dates are shown as * and ** denote significant difference (*p* < 0.05) and extremely significant difference (*p* < 0.01), respectively

The aim of the introducing of ScPDAT is to promote the synthesis of TAG. Both the FA profile and content were compared between *Scpdat* and WT. The total lipid contents were calculated by the sum of FAs after trans-esterification and GC quantification. The content of FAs in two strains was compared and is shown in Fig. 3a, and FA profile variation from the inoculation to day 4 are shown in Table 2. The results showed that *Scpadt* produced 23% more FAs than WT.

**Table 2:** Fatty acid composition of wild type and *Scpdat*

Time (day)/FA profiling (%)	0		1		2		3		4	
	WT	*Scpdat*	WT	*Scpdat*	WT	*Scpdat*	WT	*Scpdat*	WT	*Scpdat*
C16:0	22.9 ± 0.8	21.0 ± 0.7	31.2 ± 4.6	26.0 ± 2.6	20.0 ± 0.2	21.6 ± 2.6	21.4 ± 0.5	19.2 ± 0.4	21.7 ± 1.1	20.5 ± 1.0
C18:0	2.2 ± 0.6	1.7 ± 0.3	4.8 ± 0.0	3.6 ± 0.6	2.5 ± 0.2	2.1 ± 0.3	2.4 ± 0.0	1.7 ± 0.3	2.2 ± 0.1	2.1 ± 0.5
C16:1n9	7.2 ± 0.6	5.8 ± 0.1	0.8 ± 0.6	1.2 ± 0.5	7.3 ± 0.3	2.4 ± 3.1	6.7 ± 0.6	7.2 ± 0.9	6.4 ± 0.4	5.9 ± 1.1
C16:1n7	4.6 ± 1.8	3.8 ± 1.3	3.6 ± 0.9	3.0 ± 0.9	6.1 ± 0.3	3.0 ± 1.1	4.0 ± 1.5	3.7 ± 1.0	3.7 ± 1.2	3.9 ± 1.6
C18:1n9	1.0 ± 0.0	1.0 ± 0.5	1.7 ± 1.5	2.1 ± 0.5	1.9 ± 0.1	0.6 ± 0.5	2.9 ± 0.9	1.2 ± 0.5	1.9 ± 0.5	1.0 ± 0.1
C18:1n7	7.6 ± 0.7	6.3 ± 0.2	5.0 ± 2.1	4.9 ± 1.8	6.7 ± 0.1	3.9 ± 1.0	5.7 ± 0.8	4.6 ± 0.4	7.1 ± 0.9	5.0 ± 0.5
C16:2n6	2.4 ± 0.1	1.4 ± 0.2	1.4 ± 0.0	1.2 ± 0.1	1.8 ± 0.0	1.3 ± 0.3	2.4 ± 0.1	2.1 ± 0.1	2.5 ± 0.1	1.6 ± 0.2
C18:2n6	7.8 ± 0.6	5.0 ± 0.2	8.6 ± 0.2	9.6 ± 0.7	8.6 ± 0.5	8.1 ± 1.1	10.2 ± 0.8	9.8 ± 0.5	8.9 ± 0.0	6.7 ± 0.7
C16:3n6	1.5 ± 0.1	2.3 ± 0.0	1.0 ± 0.2	1.3 ± 0.2	1.3 ± 0.2	1.6 ± 0.0	1.8 ± 0.1	2.2 ± 0.1	1.6 ± 0.0	2.1 ± 0.1
C16:3n3	2.1 ± 0.9	1.5 ± 0.3	1.6 ± 0.1	2.3 ± 0.5	2.8 ± 0.3	2.4 ± 0.1	1.5 ± 0.8	1.7 ± 0.1	1.6 ± 0.9	1.5 ± 0.2
C18:3n6	7.2 ± 0.1	7.7 ± 0.4	6.2 ± 0.3	5.8 ± 1.1	7.4 ± 0.2	6.5 ± 0.7	6.8 ± 0.3	7.2 ± 0.2	7.5 ± 0.7	7.1 ± 0.1
C18:3n3	20.7 ± 1.8	25.2 ± 1.0	22.5 ± 0.9	25.3 ± 1.9	19.6 ± 0.5	28.8 ± 1.4	20.4 ± 1.5	23.0 ± 1.1	21.2 ± 2.7	24.9 ± 1.3
C16:4n3	10.3 ± 0.2	14.3 ± 0.6	9.9 ± 1.0	11.6 ± 1.5	11.0 ± 0.1	15.8 ± 0.2	11.5 ± 0.4	14.4 ± 0.6	11.7 ± 1.4	15.1 ± 0.3
C18:4n3	2.4 ± 0.5	2.8 ± 0.1	1.6 ± 1.2	2.2 ± 0.4	3.0 ± 0.1	1.9 ± 0.8	2.2 ± 0.5	2.1 ± 0.2	2.0 ± 0.3	2.6 ± 0.3

Date are shown as means $\bar{x}$ ± SD (*n* = 3); It is indicated that during the culture period of 0–4 days; Data of fatty acids were the percentage of peak area

The contents of saturated fatty acids (SFA), monounsaturated fatty acids (MUFA) and polyunsaturated fatty acids (PUFA) in WT and *Scpdat* are shown in Fig. 3b. Comparing both strains, there was no significant difference in the content of MSFA. However, the highly significant difference (*p* < 0.01) existed in PUFA between *Scpdat* and wild type. In fact, PUFA accounted for 83% increase of TFA from *Scpdat* to WT. Besides, significant difference (*p* < 0.05) also existed in SFA content.

**Fig. 4:**
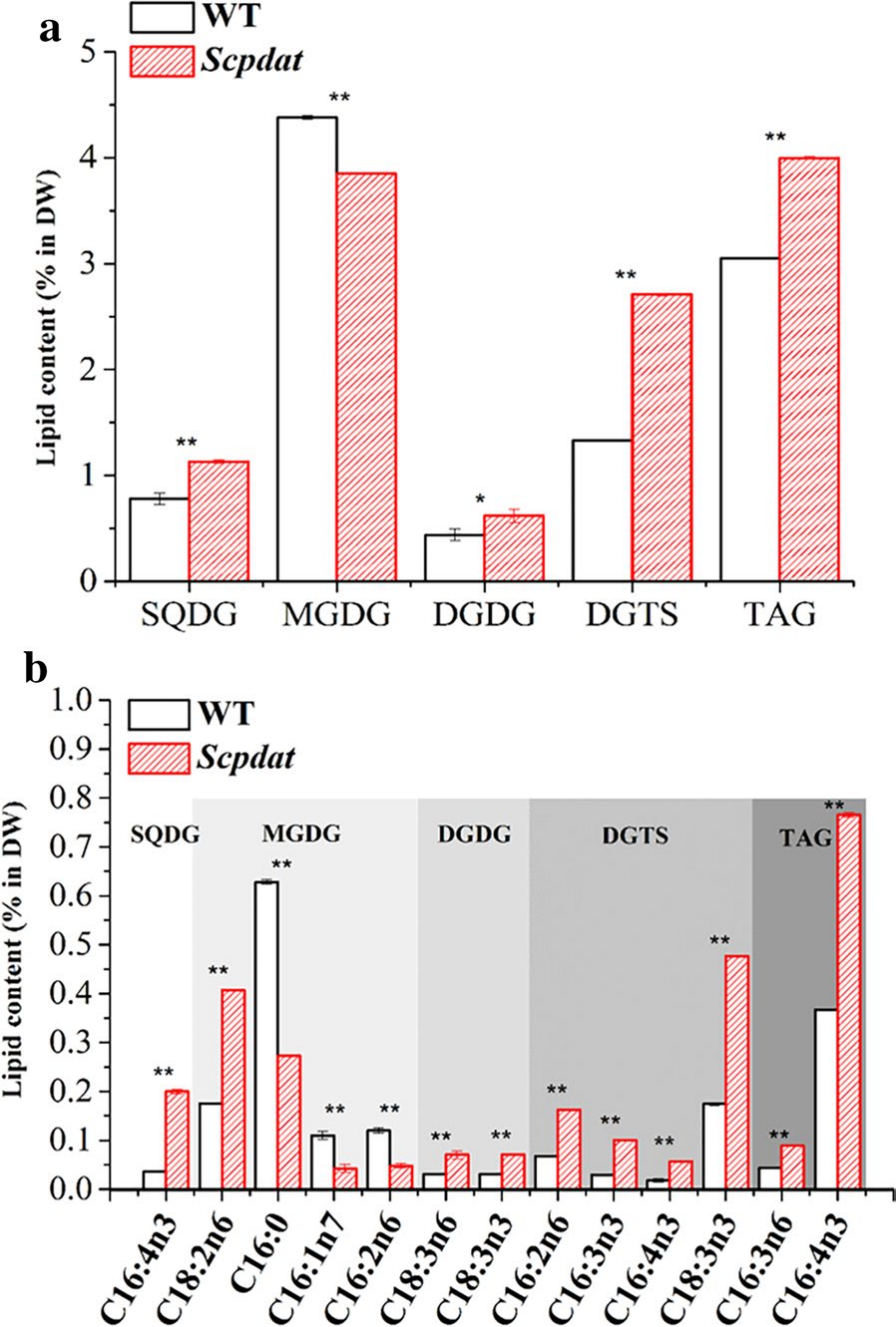
The effects on lipids of *Scpdat*. **a**. The contents of lipids in wild type and *Scpdat*. The main five esters (SQDG, MGDG, DGDG, DGTS, and TAG) were isolated by TLC method. Date are shown as * and ** denote significant difference (*p* < 0.05) and extremely significant difference (*p* < 0.01), respectively. **b** The contents of fatty acids with significant difference of SQDG, MGDG, DGDG, DGTS and TAG. The graph is summarized by GC analysis of five kinds of esters, and the left and right columns in each ester represent the wild type and *Scpdat*, respectively. Dates are shown as ** denote extremely significant difference (*p* < 0.01)

To inspect the contribution of different lipids to the increased TFA, the samples of day 3 were used to make a detailed FA analysis based on the separation of lipids by TLC. According to the character of lipid construction of *C. reinhardtii*, five types of lipids were analyzed, including sulfoquinovosyldiacylglycerol (SQDG), digalactosyldiacylglycerol (DGDG), monogalactosyldiacylglycerol (MGDG), diacylglyceroltrimethyhomoserine (DGTS), and TAG. The content of above lipids is shown in Fig. 4a and the FA content in each lipid is listed in Table 3. *Scpdat* owned significant higher amount of SQDG, DGTS and TAG (*p* < 0.01) than WT and 32% increase of TAG was achieved in *Scpdat*.

**Table 3:** Fatty acid composition of SQDG, DGDG, DGTS, MGDG and TAG in wild type and *Scpdat*

Time (day)/FA profiling (%)	MGDG		DGTS		SQDG		DGDG		TAG	
	WT	*Scpdat*	WT	*Scpdat*	WT	*Scpdat*	WT	*Scpdat*	WT	*Scpdat*
C16:0	0.63 ± 0.00	0.27 ± 0.01	0.44 ± 0.01	0.82 ± 0.00	0.36 ± 0.01	0.47 ± 0.00	0.15 ± 0.01	0.23 ± 0.01	0.38 ± 0.00	0.36 ± 0.00
C16:1n9	0.04 ± 0.00	0.02 ± 0.00	0.02 ± 0.00	0.05 ± 0.00	0.01 ± 0.00	0.01 ± 0.00	0.01 ± 0.00	0.01 ± 0.00	0.76 ± 0.00	0.87 ± 0.01
C16:1n7	0.11 ± 0.00	0.04 ± 0.00	0.03 ± 0.00	0.03 ± 0.00	0.03 ± 0.01	0.03 ± 0.01	0.02 ± 0.00	0.02 ± 0.00	0.09 ± 0.01	0.08 ± 0.01
C16:2n6	0.12 ± 0.00	0.05 ± 0.01	0.07 ± 0.00	0.16 ± 0.00	0.01 ± 0.02	0.03 ± 0.01	0.01 ± 0.01	0.01 ± 0.01	0.05 ± 0.02	0.04 ± 0.02
C16:3n6	0.11 ± 0.00	0.11 ± 0.00	0.00 ± 0.00	0.01 ± 0.00	0.01 ± 0.01	0.03 ± 0.00	0.00 ± 0.00	0.00 ± 0.00	0.04 ± 0.00	0.09 ± 0.00
C16:3n3	0.06 ± 0.01	0.04 ± 0.00	0.03 ± 0.00	0.10 ± 0.00	0.01 ± 0.01	0.02 ± 0.00	0.00 ± 0.00	0.00 ± 0.00	0.02 ± 0.00	0.03 ± 0.01
C16:4n3	0.59 ± 0.00	0.69 ± 0.01	0.02 ± 0.00	0.06 ± 0.00	0.04 ± 0.01	0.20 ± 0.00	0.01 ± 0.01	0.00 ± 0.01	0.37 ± 0.00	0.77 ± 0.00
C18:0	0.14 ± 0.00	0.10 ± 0.00	0.03 ± 0.01	0.06 ± 0.00	0.04 ± 0.01	0.04 ± 0.01	0.03 ± 0.00	0.04 ± 0.00	0.19 ± 0.00	0.21 ± 0.00
C18:1n9	0.17 ± 0.00	0.12 ± 0.00	0.10 ± 0.00	0.17 ± 0.00	0.06 ± 0.01	0.06 ± 0.01	0.06 ± 0.01	0.05 ± 0.01	0.12 ± 0.00	0.13 ± 0.00
C18:1n7	0.17 ± 0.00	0.09 ± 0.00	0.09 ± 0.00	0.14 ± 0.00	0.04 ± 0.00	0.04 ± 0.01	0.02 ± 0.00	0.03 ± 0.01	0.13 ± 0.00	0.12 ± 0.00
C18:2n6	0.53 ± 0.00	0.46 ± 0.00	0.18 ± 0.00	0.41 ± 0.00	0.04 ± 0.01	0.07 ± 0.01	0.04 ± 0.00	0.07 ± 0.01	0.20 ± 0.01	0.25 ± 0.01
C18:3n6	0.23 ± 0.00	0.13 ± 0.00	0.10 ± 0.00	0.18 ± 0.00	0.04 ± 0.01	0.03 ± 0.01	0.03 ± 0.01	0.07 ± 0.01	0.23 ± 0.00	0.31 ± 0.00
C18:3n3	1.36 ± 0.00	1.65 ± 0.00	0.17 ± 0.00	0.48 ± 0.00	0.06 ± 0.01	0.09 ± 0.00	0.03 ± 0.01	0.07 ± 0.00	0.39 ± 0.00	0.64 ± 0.00
C18:4n3	0.17 ± 0.02	0.10 ± 0.02	0.08 ± 0.02	0.11 ± 0.01	0.04 ± 0.01	0.04 ± 0.01	0.05 ± 0.01	0.05 ± 0.02	0.09 ± 0.01	0.15 ± 0.01

Date are shown as means $\bar{x}$  ± SD (*n* = 3). Sample of wild type and *Scpdat* were cultured at the 3rd day. Data of fatty acids were the percentage of DW

In terms of FAs shown in Fig. 4b, the PUFAs in SQDG (C16:4n3), DGDG (C18:3n3 and C18:3n6), DGTS (C16:2n6, C16:3n3, C16:4n3 and C18:3n6) and TAG (C16:3n6 and C16:4n3) of *Scpdat* were highly significantly (*p* < 0.01) increased, and contributed to the increase of TFA. On the contrast, the major C16 series FAs highly significantly (*p* < 0.01) decreased in MGDG of *Scpdat* and only C18:2n6 increased (*p* < 0.01).

## Discussion

The synchronization of the growth and TAG is an ideal route for the sustainable production of biofuels; however, the conflict between the growth and energy storage compound’s accumulation is endogenous. As a redundant system, the reassembly of the biomass to retard the impact of stress remains in the nature of the cell. So, there were few successes in the establishment of engineered microalgae strains by way of enhancing the expression of enzymes involving in biosynthesis of FAs or lipids, e.g., acetyl-CoA carboxylases (ACCases), whose over-expression did not promote the simultaneous improvement of TAG with the growth [17, 18], or by modification of expression regulation, e.g., by transcription factors or promoters [19].

The chimeric promoter Hsp70-RBCS2 has been proved as a good promoter for the exogenous gene expression in the microalgae cells targeting to the high expression [20, 21]. The high temperature, higher than 35 °C, is commonly used to induce the expression with the aid of Hsp70 promoter [20, 22, 23]. It is interesting here that under the convenient temperature, 25 °C, the expression of the chimeric ScPDAT consisted to the growth, and with a slight effect to the growth, 22% increase in total fatty acid and 32% increase in TAG was obtained. The effects on FAs were also inspected under nitrogen-absent cultivation (unpublished data); however, the enhancement of FAs was only shown within the first 24 h, 10% increase (percentage of DW) in *Scpdat* and 10% decrease in WT. It is postulated that CC-137 mainly accumulates starch under the beginning of nitrogen-absent conditions, and the effect of ScPDAT on FAs was masked by significant changes in overall carbon flux.

The significantly increased FAs in TAG were two C16 species, C16:3n6 and C16:4n3, which were all prokaryotic FAs in chloroplast, while the significantly decrease FAs in MGDG were also C16 FAs. CrPDAT has been shown with a strong in vitro activity to utilize MGDG as the acyl donor to synthesize TAG, and mediates the chloroplast membrane turnover and degradation [10]. It is reasonable to postulate that exogenous ScPDAT, with high similarity to CrPDAT, converted the MGDG in the chloroplast directly to TAG on a certain degree. This conversion of MGDG caused sequential decrease in the photosynthesis ability indicated by the decrease of *F*_v_/*F*_m_.

Furthermore, C16:4n3 and C18:3n3 were two chloroplast biomarker FAs, which were mostly existing in the chloroplast [24], and were also the major contributors to the increased lipid content. Combining *F*_v_/*F*_m_ changes and FAs profile changes in different lipids (Fig. 4b, Additional file 3: Figure S3), especially those of C16 series FAs, it can be postulated that exogenous ScPDAT mainly affected the FA and lipid metabolism in chloroplast, with the help of CrPDAT’s cTP. However, the chloroplast location of cTP-fused ScPDAT needs more direct proofs, and it will be carried out by way of fluorescence protein labeling as Yang et al. [25] or Mori et al.’s [26] reports.

It is interesting that the exogenous ScPDAT showed a growth-related expression monitored by western blot. The similar pattern was also found in the expression of endogenous chloroplast glyceraldehyde-3-phosphate dehydrogenase (GAP3, chloroplast GAPDH of *C. reinhardtii*, phytozome: Cre01.g010900.t1.2) (data were not shown here). The regulation of Hsp70-RBCS2 promoter-driven expression has already reported to be manipulated on both transcription and RNA silence levels. Because both ScPDAT and GAP3 were expressed with cTPs, whether the growth-related expression is the interaction between cTPs and Hsp70-RBCS2 promoter will be further studied and verified on both transcript and protein levels. The understanding of the expression regulation will contribute to the rational design of microalgae based expression system.

## Conclusion

A CrPDAT cTP-fused ScPDAT in the upstream was constructed in the pChlamy vector and the transformants of *C. reinhardtii* CC-137 were screened by hygromycin B and PCR verification. On the growth, *Scpdat* showed a slow initial growth, but recovered quick and reached to the same final level as WT, while a slight decrease of *F*_v_/*F*_m_, was also observed during the cultivation. The result of western blot disclosed that the abundance of ScPDAT protein changed with the growth, which reached to the peak during the exponential growth phase. Both the fatty acid profile and lipid content analysis showed that the total fatty acid and TAG accumulation in *Scpdat* increased by 22 and 32%, respectively. The shift of chloroplast lipids metabolism was considered as the reason for the delay of the initial growth and the decrease in *F*_v_/*F*_m_. In summary, this report showed a simultaneous product of both lipid and biomass without stress induction, and will be a potential solution for the biofuel production in the future.

## Methods

### Microalgal strain and growth conditions

Microalgal strain *C. reinhardtii* CC-137 was obtained from the Chlamydomonas Resource Center with the ID of CC-137. Microalgae were grown as batch cultures in flasks with tris acetate phosphate (TAP) media. Cultures in liquid medium or on the plate were grown at 25 ± 1 °C in an artificial climate incubator, under a 24-h light photoperiod provided by cool white fluorescence light with 50-μmol photons m^−2^ s^−1^ irradiance. For liquid culture, 100-mL culture was incubated in 250-mL conical flasks.

### Gene cloning and analysis of ScPDAT

Fused with 279 bp transmembrane signal peptide from CrPDAT (chloroplast transit peptide), 1953 bp coding region of ScPDAT was cloned into the pChlamy expression vector (GeneArt^®^ Chlamydomonas TOPO^®^ Engineering Kits, Invitrogen) through the RF cloning method [27] as following: ScPDAT (6 nM), pChlamy vector plasmid (300 pM), HS DNA premixed polymerase (Takara). The products of RF cloning were transformed into DH5α cell. The pChlamy–ScPDAT plasmid was sequenced (Fig. 2) by Takara for verification.

### Transformation of pChlamy–ScPDAT into microalgae

The recombinant plasmid pChlamy–ScPDAT was electroporated into microalgae using a Bio-Rad apparatus, following the kit’s protocol. Specifically, after linearized by *pvu*I restriction enzyme (Takara), 2–3 µg pChlamy–ScPDAT and the same volume deionized water as a comparison were used per electroporation.

### Selecting the engineered microalgae

The transformed algal cells in the 6-well plate were harvested and cultured into the solid selection medium supplemented with 10-μg/mL hygromycin (Invitrogen). The surviving colonies were picked up and grown in liquid medium with hygromycin B and subcultured every week. To preclude the impact of hygromycin in engineered microalgae, cells were cultured in TAP medium without hygromycin for three culture cycles prior to biochemical and molecular analyses and PCR verification of transgene was performed before further cultivation using universal primers of the pChlamy vector and specific primers of ScPDAT, respectively.

### Western blot test of the expression of ScPDAT

To examine the expression of ScPDAT protein in the engineered microalgae, western blot analysis was performed. The anti-His antibody (ABclonal) was used to detect the ScPDAT proteins. Briefly, 50 mL cultivation of cells was collected by centrifuge at 4 °C, 10,000*g* for 5 min. After removal of the supernatant, cells were frozen in liquid nitrogen, and stored in − 80 °C refrigerator.

Before the lysis of cells, 20–30 mL of ethanol per sample was used to extract the algae pigment following a centrifuge at 4 °C, 10,000*g* for 5 min. Then cells were washed by cold deionized water twice to remove ethanol. Adding 1 mL of lysis buffer (50 mM Tris–HCl, pH 7.5, 0.15 M NaCl, 1 mM EDTA, 1% NP-40, 10% glycerol, and 1 mM phenylmethylsulfonyl fluoride-PMSF and complete protease inhibitor cocktails) per sample and performed further lysis process on ice [28].

The algal cells were disrupted on a tissue disintegrator (SCIENTZ-48) and placed on ice for 1–2 h. After centrifugation at 12,000*g* for 20 min, the supernatant was assayed for protein concentration. Uniform protein content was applied to SDS-PAGE electrophoresis. An aliquot of 25 μg protein from each sample was resolved by 10% SDS-PAGE and stained with Coomassie Brilliant Blue G-250 to visualize the protein bands. The other identical gel was electrotransferred to a PVDF membrane for standard western blot analysis. Two SDS-PAGE gels of the same sample were transplanted and incubated with His antibody and GAPDH (CST) antibody, respectively. The GAPDH antibody was used as internal control at a dilution of 1:1000. Finally, The PVDF membrane immersed in the exposure liquid (Tanon™ High-sig ECL Western Blotting Substrate) developed on the exposure instrument (Multifunctional imager: FUSION-FX5-820).

### Growth and chlorophyll fluorescence measurement

The microalgal concentration was determined daily by optical density measurements at 750 nm by a UV–Vis spectrophotometer (Jasco-V530). The maximum quantum efficiency of photosystem II (PS II), termed *F*_v_/*F*_m_. *F*_v_/*F*_m,_ was measured by a chlorophyll fluorometer (Water-PAM WALZ) [29, 30].

### Fatty acid and lipid content analysis

The dry cells were used to detect the fatty acid profiles or contents, and TAG contents [31, 32]. Briefly, approximately 5 mg of dry cells was weighed (MSE125P-1CE-DI, Sartorius), 5 mL 2% H_2_SO_4_–methanol (v/v H_2_SO_4_/methanol) was added, and the mixture was heated at 70 °C for 1 h. FAMEs were extracted by hexane and quantified using an Agilent GC 7890A fitted with FID and a DB-23 column (Agilent Technologies) [31]. Methyl heptadecanoate (C17, Sigma-Aldrich) was used as an internal standard to determine fatty acid recovery for quantification.

TAG quantification required total lipid extraction from biomass [33] and lipid separation by TLC. Methanol:chloroform:water (1:1:0.9) was used as the extraction solvent. A 950-µL extraction solvent composed of methanol:chloroform:water (1:2:0.8) was first added to pre-weighed dry cells with a pre-addition of the methyl heptadecanoate TAG 51:0 followed by 30 min sonication. After complete mixing, an additional 250 µL of chloroform was mixed into the solvent, followed by subsequent addition of 250 µL H_2_O. The samples were vortexed and centrifuged and then the organic phase of the lower layer was transferred to a 2-mL vial. This process was repeated two more times. The extracted lipids were pooled and then dried under a nitrogen flow. Next, 100 µL of chloroform was added to re-dissolve the total lipids, and the lipid extracts were deposited onto a TLC plate (TLC silica gel 60 F254, Merck KGA). The TLC plate was developed with hexane/diethyl ether/acetic acid (85:15:1, v/v/v), and the lipids were revealed by spraying with 0.05% (m/v) primuline (Sigma Aldrich) in acetone/water (80/20, v/v). The silica-containing TAG was scrapped off, followed by trans-esterification and GC detection.

1 $$ T_{\text{i}} \, = \,{{X_{\text{i}} } \mathord{\left/ {\vphantom {{X_{\text{i}} } {\sum {X_{\text{i}} \, \times \,100\% } }}} \right. \kern-0pt} {\sum {X_{\text{i}} \, \times \,100\% } }} $$

2 $$ M_{\text{i}} \, = \,{{\left( {{{X_{\text{i}} } \mathord{\left/ {\vphantom {{X_{\text{i}} } {X_{\text{s}} \times M_{\text{s}} }}} \right. \kern-0pt} {X_{\text{s}} \times M_{\text{s}} }}} \right)} \mathord{\left/ {\vphantom {{\left( {{{X_{\text{i}} } \mathord{\left/ {\vphantom {{X_{\text{i}} } {X_{\text{s}} \times M_{\text{s}} }}} \right. \kern-0pt} {X_{\text{s}} \times M_{\text{s}} }}} \right)} {M_{\text{a}} \, \times \,100\% }}} \right. \kern-0pt} {M_{\text{a}} \, \times \,100\% }} $$

3 $$ M_{\text{TAG}} \, = {{\left( {{{\sum {X_{\text{i}} } } \mathord{\left/ {\vphantom {{\sum {X_{\text{i}} } } {X_{\text{s}} \, \times \,M_{\text{s}} }}} \right. \kern-0pt} {X_{\text{s}} \, \times \,M_{\text{s}} }}} \right)} \mathord{\left/ {\vphantom {{\left( {{{\sum {X_{\text{i}} } } \mathord{\left/ {\vphantom {{\sum {X_{\text{i}} } } {X_{\text{s}} \, \times \,M_{\text{s}} }}} \right. \kern-0pt} {X_{\text{s}} \, \times \,M_{\text{s}} }}} \right)} {M_{\text{a}} \, \times \,100\% }}} \right. \kern-0pt} {M_{\text{a}} \, \times \,100\% }}\,, $$

where *T*_i_, *M*_i_ and *M*_TAG_ were the individual fatty acid percentage of total fatty acids, fatty acid content and TAG content, respectively [32].

## Additional files

**Additional file 1: Figure S1.** The alignment of PDATs. The protein sequences of CrPDAT (GenBank:AFB73928), ScPDAT (GenBank:NM_001183185), AtPDAT (GenBank:At5g13640), LPLA2 (GenBank:NM_012320.3) and LCAT (GenBank:NG_009778.1) were aligned by Clustalw2 and Espript 3.0 software. ※: Catalytic triad, ●: disulfide bond, ☆: conserved tyrosine, ━: GAP1 region, ┉: GAP2 region, △: head group related to acyl donor, □: lid loop. Homology comparison of PDAT sequences with different sources; the results showed that PDAT had higher similarity with human of LCAT and LPLA2. The LCAT is responsible for transferring the acyl phosphate from the phospholipids to cholesterol; LPLA2 is responsible for transferring the acyl of phospholipids to *N*-acetyl-d-sphingosine. Because of the functional similarity between PDAT and LCAT/LPLA2, phospholipids can be used as acyl donors, PDAT catalytic mechanism can be predicted based on homologous sequence comparison.

**Additional file 2: Figure S2.** Verification of the Chlamy–ScPDAT plasmid and the *Scpdat*. (A). Lanes 1,2: the PCR products amplified with the carrier universal primers and ScPDAT-specific primers; (B). Lane: the *Scpdat* clone verified by the specific primers.

**Additional file 3: Figure S3.** The synthesis of C16 series FA in *C. reinhardtii.*

## Abbreviations

FA: fatty acid
TAG: glyceryl trioleate
PDAT: phospholipid: diacylglycerol acyltransferase
DGAT: diacylglycerol acyltransferase
GPAT: glycerol-3-phosphate acyltransferase
LPAAT: lysophosphatidic acid acyltransferase
LPLA2: lysosomal phospholipase A2
LCAT: lecithin–cholesterol acyltransferase
DAG: diacylglycerol
SDP1: sugar-dependent1
PXA1: peroxisomal transporter1
ALA: α-linolenic acid
PL: phospholipid
PC: phosphatidylcholine
GAPDH: glyceraldehyde phosphate dehydrogenase
GC: gas chromatography
FID: flame ionization detector
TLC: thin-layer chromatography
PSII: photosystem II
DW: dry weight
SFA: saturated fatty acid
MUFA: monounsaturated fatty acids
PUFA: polyunsaturated fatty
MGDG: monogalactosyldiacylglycerol
DGDG: digalactosyldiacylglycerol
SQDG: sulfoquinovosyldiacylglycerol
DGTS: diacylglyceryltrimethylhomoserine
ACCase: acetyl-CoA carboxylase
FAs: fatty acid synthase
