# Supplementary figures and images for: The synchronous TAG production with the growth by the expression of chloroplast transit peptide-fused ScPDAT in Chlamydomonas reinhardtii
Source: Biotechnol Biofuels. 2018 Jun 6;11:156. doi: 10.1186/s13068-018-1160-6 (PMC5989348; doi:10.1186/s13068-018-1160-6)

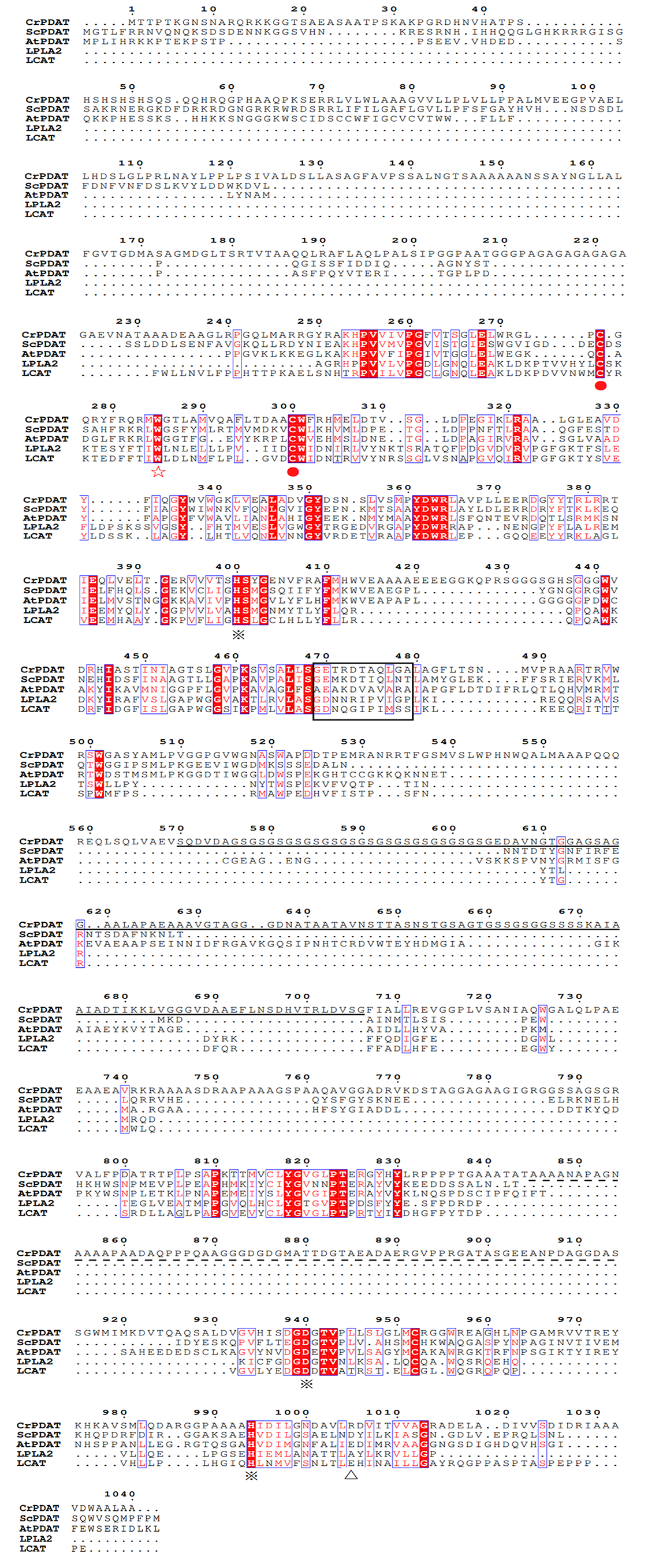

Supplement: Supplementary file 1 — Additional file 1: Figure S1. The alignment of PDATs. The protein sequences of CrPDAT (GenBank:AFB73928), ScPDAT (GenBank:NM_001183185), AtPDAT (GenBank:At5g13640), LPLA2 (GenBank:NM_012320.3) and LCAT (GenBank:NG_009778.1) were aligned by Clustalw2 and Espript 3.0 software. ※: Catalytic triad, ●: disulfide bond, ☆: conserved tyrosine, ━: GAP1 region, ┉: GAP2 region, △: head group related to acyl donor, □: lid loop. Homology comparison of PDAT sequences with different sources; the results showed that PDAT had higher similarity with human of LCAT and LPLA2. The LCAT is responsible for transferring the acyl phosphate from the phospholipids to cholesterol; LPLA2 is responsible for transferring the acyl of phospholipids to N-acetyl-d-sphingosine. Because of the functional similarity between PDAT and LCAT/LPLA2, phospholipids can be used as acyl donors, PDAT catalytic mechanism can be predicted based on homologous sequence comparison. [file 13068_2018_1160_MOESM1_ESM.tif]

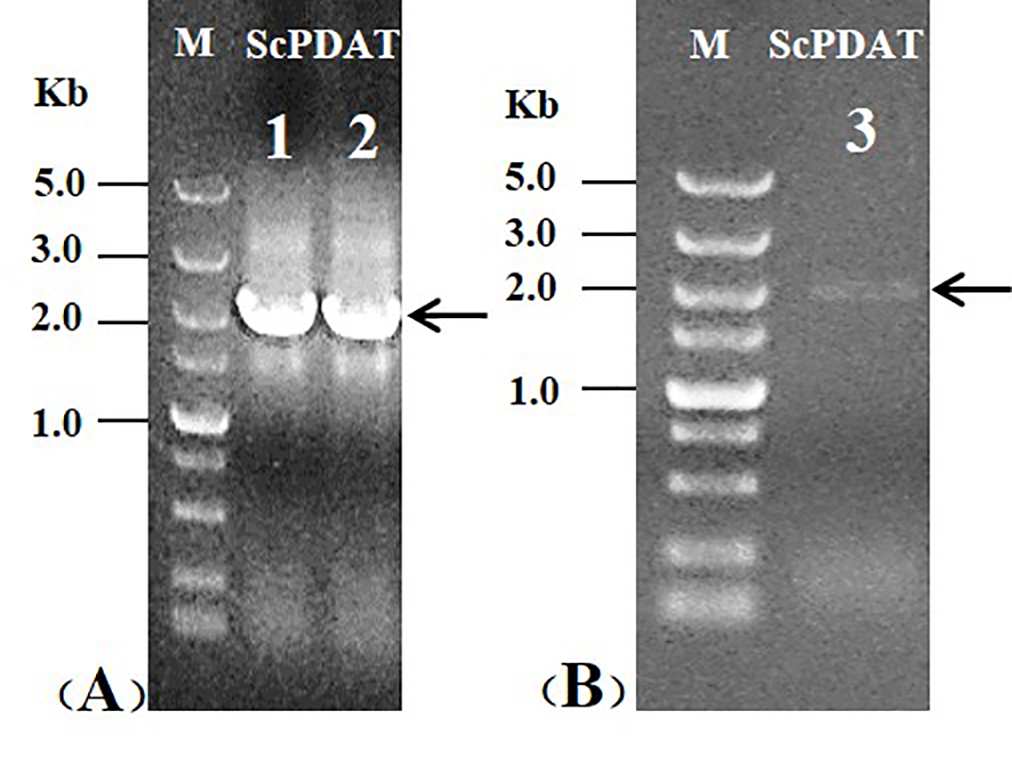

Supplement: Supplementary file 2 — Additional file 2: Figure S2. Verification of the Chlamy–ScPDAT plasmid and the Scpdat. (A). Lanes 1,2: the PCR products amplified with the carrier universal primers and ScPDAT-specific primers; (B). Lane: the Scpdat clone verified by the specific primers. [file 13068_2018_1160_MOESM2_ESM.tif]
